# Weight loss effects of methotrexate and cyclophosphamide

**DOI:** 10.18632/oncotarget.14569

**Published:** 2017-01-10

**Authors:** Dave Bridges

**Affiliations:** Department of Nutritional Sciences, University of Michigan School of Public Health, Ann Arbor, MI, USA

**Keywords:** energy balance, chemotherapy, obesity, adipogenesis, methotrexate

The pro-oncogenic effects of obesity have led to a dramatic rise in obesity-associated cancers including breast, ovarian, prostate, and gastrointestinal cancers [1]. Confounding the treatment of these and other patients is different physiological responses to chemotherapeutic agents in lean and obese subjects. In this issue, Myers *et al.* describe an anti-obesity effect of the commonly prescribed drugs methotrexate and cyclophosphamide on obesity in mice. These drugs act as anti-folate and DNA alkylating agents [2, 3], but in spite of their different mechanisms of action, both cause substantial weight loss at sub-lethal doses. While cancer survivorship is often associated with increased adiposity, the mechanisms underlying this are unclear [4, 5]. Disentangling the metabolic effects of the cancer from the treatment is especially difficult for chemotherapeutic drugs. In the case of methotrexate though, its wide prescription for immunological diseases may lend some clues in to how these drugs affect energy balance independent from malignancies.

This study leads to several interesting future questions, specifically in how these drugs function to reduce overweight and obesity. The loss of adiposity indicates negative energy balance, the source of which was not directly identified in this study. One possibility is that energy expenditure is increased outside of the window measured in this study (the first few days of treatment). This would be consistent with direct effects of methotrexate on uncoupled oxygen consumption in HepG2 cells. Whether this is true of other energy dissipating tissues, and how this occurs is a key next step. Another related question is the impact of these drugs on muscle mass and quality, as some cancers and cancer-associated therapies such as glucocorticoids cause muscle wasting, which in turn can cause alterations in energy balance [6]. The authors also show the depletion of adipogenic precursors in obese mice treated with cyclophosphamide or methotrexate. Without changes in energy balance, a lack of adipogenic ability would likely lead to ectopic lipid storage, for example in the liver, but the authors show a decrease in hepatosteatosis in this model. While it is unsurprising that these drugs target dividing cells such as adipogenic precursors, this may have long term effects on fat storage and metabolism especially in the context of post-chemotherapy survivorship. This is especially important as chronic changes in metabolism caused by chemotherapy may underlie some of the increased risk of metabolic syndrome in cancer survivors [4].
